# Hermite–Hadamard inequalities and their applications

**DOI:** 10.1186/s13660-018-1895-4

**Published:** 2018-11-12

**Authors:** Marcela V. Mihai, Muhammad Uzair Awan, Muhammad Aslam Noor, Jong Kyu Kim, Khalida Inayat Noor

**Affiliations:** 1 Department Scientific-Methodical Sessions, Romanian Mathematical Society-Branch Bucharest, Bucharest, Romania; 2 Department of Mathematics, GC University, Faisalabad, Pakistan; 3 Department of Mathematics, COMSATS University Islamabad, Islamabad, Pakistan; 4 Department of Mathematics Education, Kyungnam University, Changwon, Korea

**Keywords:** 26D15, 26A51, log-convex function, Hermite–Hadamard inequalities, Generalized logarithmic means

## Abstract

New Hermite–Hadamard type inequalities are established. Some corresponding examples are also discussed in detail.

## Introduction and preliminaries

In recent years, much attention has been given by many researchers to theory of convexity because of its great utility in various fields of pure and applied sciences. The theories of convex functions and inequalities are closely intertwined. A very interesting inequality, which is extensively studied in the literature, is due to Hermite and Hadamard who discovered it independently; now it is known as Hermite–Hadamard inequality. It provides a necessary and sufficient condition for a function to be convex. This famous result of Hermite and Hadamard reads as follows:

Let $\mathcal{F}:I\subset\mathbb{R}\rightarrow\mathbb{R}$ be a convex function, where $a,b\in I$ with $a< b$. Then

1.1 $$ \mathcal{F} \biggl(\frac{a+b}{2} \biggr)\leq\frac{1}{b-a} \int_{a}^{b} \mathcal{F}(x)\,\mathrm{d}x\leq \frac{\mathcal{F}(a)+\mathcal{F}(b)}{2}. $$

For details and applications, see [3, 4].

In this paper, we consider the classes of convex, log-convex and log-concave functions. We derive some new Hermite–Hadamard type inequalities for such functions in connection with generalized logarithmic means. We also discuss some special cases.

### Theorem 1.1

([4, 5])

*Let* *μ* *be a Borel probability measure on* $[a,b]$. *Then every convex function* $\mathcal{F}:I\supseteq [a,b]\to\mathbb{R}$ *satisfies the following analogue of Hermite–Hadamard inequality*:

$$\mathcal{F}(b_{\mu})\leq \int_{a}^{b}\mathcal{F}(x)\,\mathrm{d}\mu \leq \frac{b-b_{\mu}}{b-a}\mathcal{F}(a) +\frac{b_{\mu }-a}{b-a}\mathcal{F}(b). $$

In an article from 1995, C. E. M. Pearce and J. E. Pečarić [7], formulated a result of Jensen type, considering generalized logarithmic means of Stolarsky [9]. For $a,b>0$, two distinct numbers, these means are defined by the formulas

$$L_{p} ( a,b ) = \textstyle\begin{cases} [ \frac{b^{p}-a^{p}}{p(b-a)} ] ^{1/(p-1)}, & p\neq0,1,\\ \frac{b-a}{\log b-\log a}, & p=0,\\ \frac{1}{e} [ \frac{b^{b}}{a^{a}} ] ^{1/(b-a)}, &p=1, \end{cases} $$

and for $a=b>0$ we have $L_{p}(a,a)=a$.

In mathematics, the polylogarithm (also known as Jonquière function, for Alfred Jonquière) is a special function of order *s* and argument *z* given by

$$\mathit{Li}_{s}(z)=\sum_{k=1}^{\infty} \frac{z^{k}}{k^{s}}, $$

where $z\in\mathbb{C}$. Only for special values of *s* does the polylogarithm reduce to an elementary function such as the natural logarithm or rational functions. The reflection formula was already published by Landen in 1760, prior to its appearance in a book written in 1768 by Euler ([2], Section 10); an equivalent to Abel’s identity was already published by Spence in 1809, before Abel wrote his manuscript in 1826 ([10], Section 2). Bilogarithmic functions were introduced by Carl Johan Danielsson Hill in 1828. Don Zagier in 1989 remarked that the dilogarithm is the only mathematical function possessing a sense of humor. Some particular cases of the function are:

$$\begin{aligned} &\mathit{Li}_{-3}(x)=\frac{x (1+4x+x^{2} ) }{(1-x)^{4}}, \qquad \mathit{Li}_{-2}(x)=\frac{x(x+1)}{(1-x)^{3}},\quad x\in\mathbb{R} \setminus \lbrace1 \rbrace; \\ &\mathit{Li}_{-1}(x)=\frac{x}{(1-x)^{2}},\qquad \mathit{Li}_{0}(x)= \frac{x}{1-x},\quad x\in\mathbb{R}\setminus \lbrace1 \rbrace; \\ &\mathit{Li}_{1}(x)=-\log(1-x),\quad x\in (-\infty, 1 ). \end{aligned}$$

**Figure 1:**
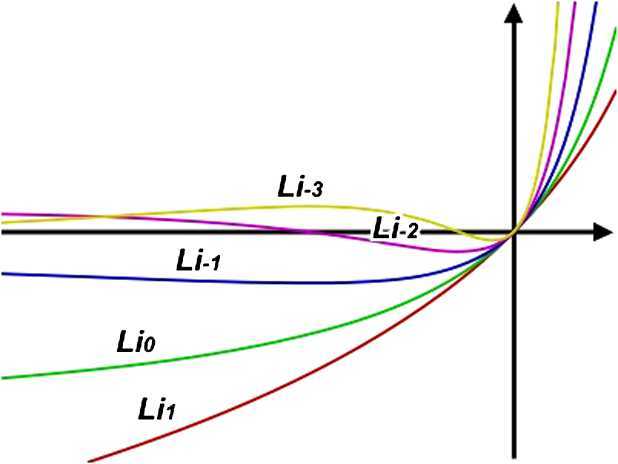
Jonquière functions in some particular cases

These functions are represented graphically in Fig. 1.

Other special functions are Struve functions, denoted as $H_{\alpha }(x)$. These are the solutions of the non-homogeneous Bessel’s differential equation

$$ x^{2}\frac{\mathrm{d}^{2}y}{\mathrm{d}x^{2}}+x\frac{\mathrm {d}y}{\mathrm{d}x}+ \bigl(x^{2}-a^{2} \bigr)y=\frac{4 ( \frac{x}{2} )^{\alpha+1} }{\sqrt{\pi}\varGamma ( \alpha+\frac{1}{2} ) }, $$

introduced by Hermann Struve in 1882. The complex number *α* is the order of the Struve function, and is often an integer. Struve functions have the following power series:

$$ H_{\alpha}(x)=\sum_{m=0}^{\infty} \frac{(-1)^{m}}{\varGamma (m+\frac{3}{2} ) \varGamma (m+\alpha+\frac{3}{2} ) } \biggl(\frac{x}{2} \biggr)^{2m+\alpha+1}, $$

where $\varGamma(z)$ is the gamma function. Another definition of the Struve function, for values of *α* satisfying $Re(z)>-\frac {1}{2}$, is possible using an integral representation

$$ H_{\alpha}(x)=\frac{2 ( \frac{x}{2} ) ^{\alpha}}{\sqrt {\pi}\varGamma (\alpha+\frac{1}{2} ) } \int_{0}^{\pi /2}\sin(x \cos t)\sin^{2\alpha}t \, \mathrm{d}t. $$

**Figure 2:**
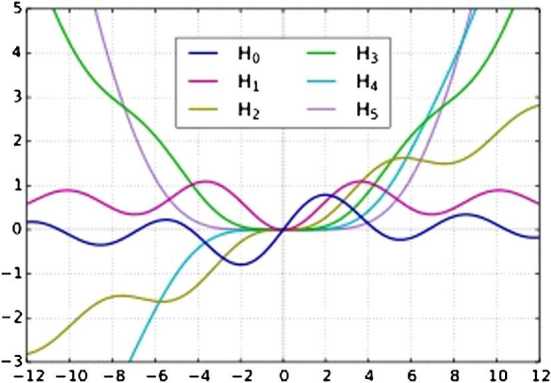
Struve functions in some particular cases

For more details, see [6]. These functions are represented graphically in Fig. 2.

## Results and discussions

We will use the result of Pearce and Pečarić to indicate analogues of Hermite–Hadamard inequalities in several special cases.

### Theorem 2.1

(C. E. M. Pearce and J. E. Pečarić [7])

*Let* $0< a< b$, $[c,d]\subset (0,1)\cup(1,+\infty)$ *and* $\mathcal{F}:[a,b]\rightarrow{}[ c,d]$ *a continuous function*. *If* $p\neq0,1$ *and* $\mathcal{G}(x)=\mathcal{F}(x^{1/(p-1)})$ *is convex*, *or* $p=1$ *and* $\mathcal{G}(x)=\mathcal{F}(e^{x})$ *is convex*, *then*

$$\mathcal{F} \bigl( L_{p}(a,b) \bigr) \leq\frac{1}{b-a} \int _{a}^{b}\mathcal{F}(x)\,\mathrm{d}x. $$

*If* $p\neq0,1$ *and* $\mathcal{G}(x)=\mathcal{F}(x^{1/(p-1)})$ *is concave*, *or* $p=1$ *and* $\mathcal{G}(x)=\mathcal{F}(e^{x})$ *is concave*, *then the inequality is reversed*.

### Proof

The proof is immediate and is reduced to Jensen’s inequality in integral form, namely, if $p\neq0,1$ and $\mathcal{G}$ is convex, we have

$$\begin{aligned} \mathcal{F} \bigl( L_{p}(a,b) \bigr) & =\mathcal{F} \biggl[ \biggl( \frac{1}{b-a} \int_{a}^{b}t^{p-1}\,\mathrm{d}t \biggr) ^{1/(p-1)} \biggr] \\ & =\mathcal{G} \biggl( \frac{1}{b-a} \int_{a}^{b}t^{p-1}\,\mathrm {d}t \biggr) \leq \frac{1}{b-a} \int_{a}^{b}\mathcal{G} \bigl(t^{p-1} \bigr) \,\mathrm{d}t=\frac{1}{b-a} \int_{a}^{b}\mathcal{F}(t)\,\mathrm{d}t. \end{aligned}$$

If $p\neq0,1$ and $\mathcal{G}$ is concave, then the inequality is reversed.

If $p=1$ and $\mathcal{G}$ is convex, then

$$\begin{aligned} \mathcal{F} \bigl( L_{1}(a,b) \bigr) & =\mathcal{F} \bigl( I(a,b) \bigr) =\mathcal{F} \biggl[ \exp \biggl(\frac{1}{b-a} \int_{a}^{b} \log t\,\mathrm{d}t \biggr) \biggr] \\ & =\mathcal{G} \biggl( \frac{1}{b-a} \int_{a}^{b}\log t\,\mathrm {d}t \biggr) \leq \frac{1}{b-a} \int_{a}^{b}\mathcal{G}(\log t)\,\mathrm{d}t = \frac{1}{b-a} \int_{a}^{b}\mathcal{F}(t)\,\mathrm{d}t. \end{aligned}$$

Similarly as above, if $\mathcal{G}$ is a concave function then the reversed inequality holds. □

### Theorem 2.2

*Let* $0< a< b$, $[c,d]\subset(0,1)\cup(1,+\infty)$ *and let* $\mathcal {F}:[a,b]\rightarrow {}[ c,d]$ *be a continuous and strictly increasing function*, *with the property that* $\mathcal{F}^{-1}$ *is log*-*concave*. *Then*

$$\mathcal{F} \bigl( I(a,b) \bigr) \leq\frac{1}{b-a} \int _{a}^{b}\mathcal{F}(x)\,\mathrm{d}x\leq \frac {L(a,b)-a}{b-a}\mathcal{F}(a)+\frac{b-L(a,b)}{b-a}\mathcal{F}(b). $$

*If the function* $\mathcal{F}$ *is strictly decreasing*, *then the last inequality is reversed*.

Left inequality was noticed by Seiffert [8] in 1989.

### Proof

Since $\mathcal{F}^{-1}$ is the first power, we consider the case of $p=1$ and $\mathcal{G}(x)=\mathcal{F} (e^{x} ) $ of Theorem 2.1. Therefore, it is sufficient to prove that the function $\mathcal{G}$ is convex for obtaining the left inequality, i.e.,

$$ \mathcal{G} \bigl((1-\lambda)x+\lambda y \bigr)=\mathcal{F} \bigl( e^{(1-\lambda )x+\lambda y} \bigr) \leq(1-\lambda)\mathcal{F} \bigl( e^{x} \bigr) +\lambda\mathcal {F} \bigl( e^{y} \bigr) =(1-\lambda)\mathcal{G}(x)+ \lambda\mathcal{G}(y), $$

which is equivalent to

2.1 $$ e^{(1-\lambda)x+\lambda y}\leq\mathcal{F}^{-1} \bigl((1-\lambda ) \mathcal{F} \bigl( e^{x} \bigr) +\lambda\mathcal{F} \bigl( e^{y} \bigr) \bigr), $$

because the function $\mathcal{F}^{-1}$ is strictly increasing. Making substitutions $\mathcal{F} ( e^{x} )=u$ and $\mathcal{F} ( e^{y} )=v$, we obtain $x=\log\mathcal {F}^{-1}(u) $ and $y=\log\mathcal{F}^{-1}(v)$ and then inequality (2.1) becomes

$$ e^{(1-\lambda)\log\mathcal{F}^{-1}(u)+\lambda\log\mathcal{F}^{-1}(v)} \leq\mathcal{F}^{-1} \bigl((1-\lambda)u +\lambda v \bigr). $$

Also

$$ (1-\lambda)\log\mathcal{F}^{-1}(u)+\lambda\log\mathcal{F}^{-1}(v) \leq\log\mathcal{F}^{-1} \bigl((1-\lambda)u +\lambda v \bigr) $$

is true because $\mathcal{F}^{-1}$ is a log-concave function. For the proof of the right-hand side inequality, we see that

$$ \frac{1}{b-a} \int_{a}^{b}\mathcal{F}(x)\,\mathrm{d}x = \frac{1}{b-a} \int_{\log a}^{\log b}\mathcal{F} \bigl( e^{u} \bigr)e^{u}\,\mathrm{d}u= \int_{\log a}^{\log b}\mathcal{F} \bigl( e^{u} \bigr) \frac {e^{u}\,\mathrm{d}u}{b-a}, $$

which is an integral of a convex function $\mathcal{F} ( e^{u} )$ with respect to the probability measure $\mathrm{d}\mu(u)=\frac{e^{u}\,\mathrm{d}u}{b-a}$. The barycenter of this measure is

$$ b_{\mu}= \int_{\log a}^{\log b}u\cdot\frac{e^{u}\,\mathrm{d}u}{b-a}=I(a,b). $$

So, according to Theorem 1.1, we have

$$\begin{aligned} \mathcal{G} (b_{\mu} )&=\mathcal{F} \bigl(e^{b_{\mu}} \bigr) = \mathcal{F} \bigl(I(a,b) \bigr) \\ &\leq \int_{\log a}^{\log b}\mathcal{G}(u)\cdot\frac{e^{u}\,\mathrm{d}u}{b-a} \leq\frac{\log b-b_{\mu}}{\log b-\log a}\mathcal{G}(\log a) +\frac{b_{\mu}-\log a}{\log b-\log a}\mathcal{G}(\log b) \\ &=\frac{L(a,b)-a}{b-a}\mathcal{F}(a)+\frac{b-L(a,b)}{b-a}\mathcal{F}(b), \end{aligned}$$

which completes the proof. □

### Example 2.3

**Figure 3:**
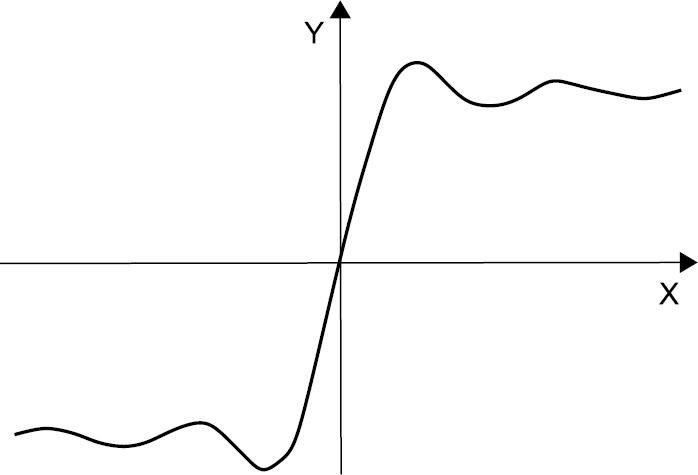
Graph of the sine integral function

The sine integral $\operatorname{Si}(x)=\int_{0}^{x}\frac{\sin t\,\mathrm{d}t}{t}$, is strictly increasing on the interval $[0,\pi]$, see Fig. 3, and $\log\operatorname{Si}(e^{x})$ is a concave function on $[0,\pi/2]$, see Niculescu and Persson [5], p. 336.

According to Theorem 2.2, for any $0\leq a< b\leq\pi/2$ the following double inequality holds:

$$\operatorname{Si} \bigl( I(a,b) \bigr) \geq\frac{1}{b-a} \int_{a}^{b}\operatorname{Si}(x) \, \mathrm{d}x\geq\frac {L(a,b)-a}{b-a}\operatorname{Si}(a)+ \frac{b-L(a,b)}{b-a}\operatorname{Si}(b). $$

### Theorem 2.4

(An analogue of Hermite–Hadamard inequality for log-convex functions)

*Let* $0< a< b$, $[c,d]\subset(0,1)\cup(1,+\infty)$ *and let* $\mathcal{F}:[a,b]\rightarrow{}[ c,d]$ *be a continuous function*, *strictly increasing and such that* $1/\mathcal{F}^{-1}$ *is convex*. *Then*

$$\mathcal{F} \bigl( L(a,b) \bigr) \leq\frac{1}{b-a} \int _{a}^{b}\mathcal{F}(x)\,\mathrm{d}x\leq \frac {bL(a,b)-ab}{ ( b-a ) L(a,b)}\mathcal{F}(a)+\frac {ab-aL(a,b)}{ ( b-a ) L(a,b)}\mathcal{F}(b). $$

*If* $\mathcal{F}$ *is strictly decreasing*, *then the last inequality holds reversed*.

The left inequality was noticed for the first time by H. Alzer [1] in 1985.

### Proof

According to Theorem 2.1, for the left inequality it will be enough to show that the function $\mathcal{G}(x)=\mathcal{F}(1/x)$ is convex, i.e.,

$$\mathcal{F} \biggl( \frac{1}{(1-\lambda)x+\lambda y} \biggr) \leq (1-\lambda)\mathcal{F} \biggl( \frac{1}{x} \biggr) +\lambda\mathcal{F} \biggl( \frac{1}{y} \biggr), $$

for any $x,y\in{}[ a,b]$ and any $\lambda\in{}[0,1]$. Let $u=\mathcal{F}(1/x)$ and $v=\mathcal{F}(1/y)$. Then the above inequality becomes

$$\frac{1}{\frac{(1-\lambda)}{\mathcal{F}^{-1}(u)}+\frac{\lambda }{\mathcal{F}^{-1}(v)}}\leq \mathcal{F}^{-1} \bigl( (1-\lambda)u+\lambda v \bigr), $$

that is,

$$\frac{1}{\mathcal{F}^{-1} ( (1-\lambda)u+\lambda v ) }\leq\frac{(1-\lambda )}{\mathcal{F}^{-1}(u)}+\frac{\lambda}{\mathcal{F}^{-1}(v)}, $$

and this is ensured by the assumption that the function $1/\mathcal {F}^{-1}$ is convex.

With this Alzer’s inequality on the left is shown.

We demonstrate both inequalities of the theorem by noting that

$$\frac{1}{b-a} \int_{a}^{b}\mathcal{F}(x)\,\mathrm{d}x = \int_{1/b}^{1/a}\mathcal{F}(1/u)\frac{\,\mathrm{d}u}{(b-a)u^{2}}, $$

which is an integral of a convex function $\mathcal{F}(1/x)$ with respect to the probability measure $\mathrm{d}\mu(t)=\frac{\mathrm{d}u}{ ( b-a ) u^{2}}$.

The barycenter of this measure is

$$b_{\mu}= \int_{1/b}^{1/a}u\frac{\mathrm{d}u}{ ( b-a) ) u^{2}}= \frac{\log b-\log a}{b-a}=1/L(a,b). $$

According to Theorem 1.1, it follows that

$$\begin{aligned} \mathcal{F} \bigl( L(a,b) \bigr) & \leq\frac{1}{b-a} \int _{a}^{b}\mathcal{F}(x)\,\mathrm{d}x= \int _{1/b}^{1/a}\mathcal{F}(1/u)\frac{\mathrm{d}u}{(b-a)u^{2}} \\ & \leq\frac{1/a-1/L(a,b)}{1/a-1/b}\mathcal{F}(a)+\frac {1/L(a,b)-1/b}{1/a-1/b}\mathcal{F}(b) \\ & =\frac{bL(a,b)-ab}{ ( b-a ) L(a,b)}\mathcal{F}(a)+\frac {ab-aL(a,b)}{ ( b-a ) L(a,b)}\mathcal{F}(b). \end{aligned}$$

The proof of the theorem is complete. □

### Example 2.5

Previous proof shows that Theorem 2.4 works for functions $\mathcal{F}$ that are continuous, strictly increasing, defined on compact intervals of strictly positive numbers and having the property that $\mathcal{G}(x)=\mathcal{F}(1/x)$ is convex.

**Figure 4:**
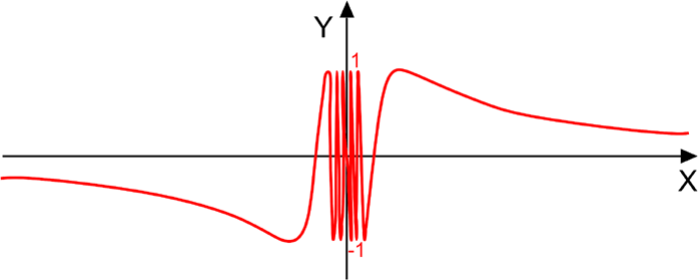
Graph of the function $\sin\frac{1}{x}$

The function $\mathcal{F}(x)=\sin(x)$ is strictly increasing on the interval $[0,\pi/2]$ and the function $\mathcal{G}(x)=\sin(1/x)$ is convex for $x\in(0,2/\pi]$, see Fig. 4. So whenever $0< a< b\leq2/\pi$, we have

$$\sin \bigl( L(a,b) \bigr)\leq\frac{1}{b-a} \int_{a}^{b}\sin x \,\mathrm{d}x \leq \frac{bL(a,b)-ab}{ ( b-a ) L(a,b)} \sin a+\frac{ab-aL(a,b)}{ ( b-a ) L(a,b)}\sin b. $$

### Theorem 2.6

*Let* $0< a< b$, $[c,d]\subset(0,1)\cup(1,+\infty)$ *and let* $\mathcal{F}:[a,b]\rightarrow{}[ c,d]$ *be a continuous function*, *strictly increasing and such that* $( \mathcal{F}^{-1} ) ^{p-1}$ *is concave*, *where* $p>1$. *Then*

$$\begin{aligned} \mathcal{F} \bigl( L_{p}(a,b) \bigr) \leq{}&\frac{1}{b-a} \int _{a}^{b}\mathcal{F}(x)\,\mathrm{d}x\\ \leq{}& \frac {b^{p-1}- ( L_{p}(a,b) )^{p-1}}{ ( b^{p-1}-a^{p-1} ) }\mathcal{F}(a) +\frac{ ( L_{p}(a,b) )^{p-1}-a^{p-1}}{ ( b^{p-1}-a^{p-1} ) }\mathcal{F}(b). \end{aligned}$$

*If* $( \mathcal{F}^{-1} ) ^{p-1}$ *is convex then the reversed inequality holds*.

### Proof

Since the argument of function $\mathcal{F}$ is $L_{p}(a, b)$, where $p>1$, we know that $p\neq1$ and consider $\mathcal{G}(x) =\mathcal{F} (x^{\frac{1}{p-1}} ) $ of Theorem 2.1. Therefore it is sufficient to prove that the function $\mathcal{G} $ is convex to obtain the left inequality, that is,

$$\begin{aligned} \mathcal{G} \bigl((1-\lambda)x+\lambda y \bigr)&=\mathcal{F} \bigl( \bigl((1- \lambda )x+\lambda y \bigr)^{\frac{1}{p-1}} \bigr) \leq(1-\lambda)\mathcal{F} \bigl(x^{\frac{1}{p-1}} \bigr) +\lambda\mathcal{F} \bigl(y^{\frac{1}{p-1}} \bigr) \\ &=(1-\lambda)\mathcal{G}(x)+\lambda\mathcal{G}(y), \end{aligned}$$

which is equivalent to

$$\bigl((1-\lambda)x+\lambda y \bigr)^{\frac{1}{p-1}} \leq\mathcal{F}^{-1} \bigl[(1-\lambda)\mathcal{F} \bigl(x^{\frac {1}{p-1}} \bigr) +\lambda\mathcal{F} \bigl(y^{\frac{1}{p-1}} \bigr) \bigr], $$

that is, because the power function exponent $p-1$ is strictly increasing, leading to

2.2 $$ (1-\lambda)x+\lambda y\leq \bigl\lbrace \mathcal{F}^{-1} \bigl[(1 -\lambda)\mathcal{F} \bigl(x^{\frac{1}{p-1}} \bigr) +\lambda\mathcal{F} \bigl(y^{\frac{1}{p-1}} \bigr) \bigr] \bigr\rbrace ^{p-1}. $$

Making substitutions $\mathcal{F} (x^{\frac{1}{p-1}} ) =u$ and $\mathcal{F} (y^{\frac{1}{p-1}} ) =v$, we obtain $x= ( \mathcal{F}^{-1}(u) ) ^{p-1}$ and $y= ( \mathcal {F}^{-1}(v) ) ^{p-1}$, where $a\leq x^{\frac{1}{p-1}}, y^{\frac{1}{p-1}}\leq b$. Due to the last inequalities and monotonicity of $\mathcal{F}^{-1}$, inequality (2.2) becomes

$$(1-\lambda) \bigl(\mathcal{F}^{-1}(u) \bigr) ^{p-1} +\lambda \bigl( \mathcal{F}^{-1}(v) \bigr) ^{p-1} \leq \bigl[ \mathcal{F}^{-1} \bigl((1-\lambda)u+\lambda v \bigr) \bigr] ^{p-1}, $$

which holds since $(\mathcal{F}^{-1} ) ^{p-1}$ is concave. For the proof of the second inequality, we see that

$$\frac{1}{b-a} \int_{a}^{b}\mathcal{F}(x)\,\mathrm{d}x = \int_{a^{p-1}}^{b^{p-1}}\mathcal{F} \bigl(u^{\frac{1}{p-1}} \bigr) \cdot\frac{u^{\frac{2-p}{p-1}} \,\mathrm{d}u}{(b-a)(p-1)}, $$

which is an integral of a concave function $\mathcal{F} (x^{\frac{1}{p-1}} ) $ with respect to the probability measure $\mathrm{d}\mu(u)=\frac{u^{\frac{2-p}{p-1}} \,\mathrm{d}u}{(b-a)(p-1)}$. The barycenter of this measure is

$$b_{\mu}= \int_{a^{p-1}}^{b^{p-1}}u\cdot\frac{u^{\frac{2-p}{p-1}} \,\mathrm{d}u}{(b-a)(p-1)}= \bigl(L_{p}(a,b) \bigr) ^{p-1}. $$

So, according to Theorem 1.1 we have

$$\begin{aligned} \mathcal{G}(b_{\mu})={}&\mathcal{F} \bigl(b_{\mu}^{\frac{1}{p-1}} \bigr) =\mathcal{F} \bigl( L_{p}(a,b) \bigr)\leq \int _{a^{p-1}}^{b^{p-1}}\mathcal{G}(x)\,\mathrm{d}\mu \leq \frac{b^{p-1}-b_{\mu}}{b^{p-1}-a^{p-1}} \mathcal{G} \bigl(a^{p-1} \bigr) \\ &{}+\frac{b_{\mu}-a^{p-1}}{b^{p-1}-a^{p-1}}\mathcal{G} \bigl(b^{p-1} \bigr)=\frac {b^{p-1}- ( L_{p}(a,b) )^{p-1}}{ ( b^{p-1}-a^{p-1} )} \mathcal{F}(a) +\frac{ ( L_{p}(a,b) )^{p-1}-a^{p-1}}{ ( b^{p-1}-a^{p-1} ) }\mathcal{F}(b), \end{aligned}$$

and the proof is complete. □

### Example 2.7

The polylogarithm function of Jonquière corresponding to $s=1$, $\mathit{Li}_{1}(x) =-\log(1-x), x\in (-\infty, 1 )$ (see Fig. 1) admits the inverse $\mathit{Li}_{1}^{-1}(x)=1-e^{-x}$, for which $( \mathit{Li}_{1}^{-1} )^{2} $ satisfies the assumptions of Theorem 2.6 on the interval $(0,1)$ and so

$$\begin{aligned} \mathit{Li}_{1} \bigl(L_{3}(a,b) \bigr) & \leq \frac{1}{b-a} \int_{a} ^{b}\mathit{Li}_{1}(x)\,\mathrm{d}x \\ & \leq\frac{b^{2}- (L_{3}(a,b) ) ^{2}}{b^{2}-a^{2}}\mathit{Li}_{1}(a) +\frac{ (L_{3}(a,b) ) ^{2}-a^{2}}{b^{2}-a^{2}}\mathit{Li}_{1}(b), \end{aligned}$$

for all $a,b\in(0,1)$.

### Example 2.8

Struve’s function corresponding to $\alpha=1$,

$$ H_{1}(x)=\frac{2x}{\pi} \int_{0}^{\pi/2}\sin(x\cos t)\sin t\,\mathrm {d}t,\quad x \in[0,3], $$

admits the inverse, for which $(H_{1}^{-1}(x) ) ^{2}$ satisfies the assumptions of Theorem 2.6 on the interval $(0,3)$ and so

$$\begin{aligned} H_{1} \bigl(L_{3}(a,b) \bigr) & \leq \frac{1}{b-a} \int_{a} ^{b}H_{1}(x)\,\mathrm{d}x \\ & \leq\frac{b^{2}- (L_{3}(a,b) ) ^{2}}{b^{2}-a^{2}}H_{1}(a) +\frac{ (L_{3}(a,b) ) ^{2}-a^{2}}{b^{2}-a^{2}}H_{1}(b), \end{aligned}$$

for all $a,b\in(0,3)$.

### Example 2.9

The polylogarithm function of Jonquière corresponding to $s=0$, $\mathit{Li}_{0}(x)=\frac{x}{1-x}, x\in\mathbb{R} \setminus \lbrace1 \rbrace$ (see Fig. 1) has the corresponding inverse $\mathit{Li}_{0}^{-1}(x)=\frac{x}{x+1}$, for which $( \mathit{Li}_{0}^{-1} )^{-1/2} $ satisfies the assumptions of Theorem 2.6 on the interval $(0.5,0.9)$ and so

$$\begin{aligned} \mathit{Li}_{0} \bigl(L_{\frac{1}{2}}(a,b) \bigr)& \leq\frac{1}{b-a} \int_{a} ^{b}\mathit{Li}_{0}(x)\,\mathrm{d}x \\ &\leq\frac{b^{-1/2}- (L_{\frac{1}{2}}(a,b) ) ^{-1/2}}{b^{-1/2}-a^{-1/2}}\mathit{Li}_{0}(a) +\frac{ (L_{\frac{1}{2}}(a,b) ) ^{-1/2}-a^{-1/2}}{b^{-1/2}-a^{-1/2}}\mathit{Li}_{0}(b), \end{aligned}$$

for all $a,b\in(0.5,0.9)$.

## Conclusion

We have established some new results of Hermite–Hadamard type for generalized logarithmic means. We have also elaborated the results with corresponding examples. Special cases were also discussed in detail. It is expected that the results of the paper will inspire interested readers.
